# A Submillimeter Continuous Variable Stiffness Catheter for Compliance Control

**DOI:** 10.1002/advs.202101290

**Published:** 2021-07-17

**Authors:** Jonas Lussi, Michael Mattmann, Semih Sevim, Fabian Grigis, Carmela De Marco, Christophe Chautems, Salvador Pané, Josep Puigmartí‐Luis, Quentin Boehler, Bradley J. Nelson

**Affiliations:** ^1^ Institute of Robotics and Intelligent Systems ETH Zurich Zurich CH‐8092 Switzerland; ^2^ Institute of Chemical and Bioengineering ETH Zurich Vladimir Prelog Weg 1 Zurich CH‐8093 Switzerland; ^3^ Departament de Ciència dels Materials i Química Física Institut de Química Teòrica i Computacional Barcelona 08028 Spain; ^4^ ICREA Catalan Institution for Research and Advanced Studies Pg. Lluís Companys 23 Barcelona 08010 Spain

**Keywords:** magnetic actuation, medical robotics, soft robotics, variable stiffness

## Abstract

Minimally invasive robotic surgery often requires functional tools that can change their compliance to adapt to the environment and surgical needs. This paper proposes a submillimeter continuous variable stiffness catheter equipped with a phase‐change alloy that has a high stiffness variation in its different states, allowing for rapid compliance control. Variable stiffness is achieved through a variable phase boundary in the alloy due to a controlled radial temperature gradient. This catheter can be safely navigated in its soft state and rigidified to the required stiffness during operation to apply a desired force at the tip. The maximal contact force that the catheter applies to tissue can be continuously modified by a factor of 400 (≈20 mN–8 N). The catheter is equipped with a magnet and a micro‐gripper to perform a fully robotic ophthalmic minimally invasive surgery on an eye phantom by means of an electromagnetic navigation system.

## Graphical abstract

In this work, a magnetically controlled catheter that is able to continuously change its stiffness is presented. Soft, magnetic tools that are enhanced with the capability to apply and control forces during surgical intervention can significantly improve outcome of the procedure. The capabilities are demonstrated by performing a fully robotic surgery for a challenging ophthalmic intervention.

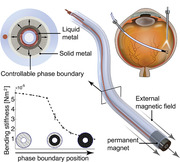

## 1 Introduction

Softness and compliance are mechanical properties highly desirable in robotic architectures and tools, particularly in those designed for applications where adaptability, shape‐morphing, delicate handling, and/or dexterity are required or advantageous. The last decade has seen an upsurge in the development of robotic systems at all scales composed of soft bodies and soft components such as end effectors,^[^ ^1^ ^]^ joints and hinges,^[^ ^2^ ^]^ and manipulators.^[^ ^3^ ^]^ For certain applications, robotic components that can gradually switch from a soft to a rigid state in which a controllable variable stiffness (VS) is displayed, could further expand the applicability of robotic tools. Nature provides countless examples of biological structures that are able to perform sophisticated tasks in complex environments through the modification of their stiffness. For example, the octopus can selectively stiffen parts of its flexible extremities to transmit high forces or to achieve complex shapes that can then be squeezed through narrow and tortuous pathways.^[^ ^4^ ^]^ Bioinspired robotic systems developed over the last decade already demonstrate the potential of VS actuation for several applications, including minimally invasive surgery (MIS).^[^ ^4^, ^5^, ^6^ ^]^ For example, in colonoscopy, VS is well established and can reduce both the time of the procedure and patient discomfort, particularly in difficult cases that require high maneuverability.^[^ ^7^, ^8^, ^9^ ^]^ However, the use of VS actuation for medical robotic applications has mainly been focused on achieving a controllable discreet binary output, directly switching from a soft to a stiff state.

Low‐melting point alloys (LMPAs), a class of fusible alloys that melt at relatively low temperatures (*T*_m_ = 41.5–98 °C), have recently been considered for the field of soft robotics and actuators.^[^ ^10^ ^]^ They become highly deformable when liquified using external heat stimuli and return to their rigid state by cooling. The deformation of LMPA components is not limited by the size of the LMPA structure in its liquid state, which makes these materials attractive substances for actuation at different scales and easily processable and compatible with other manufacturing approaches.^[^ ^11^ ^]^ An advantage of these alloys over other thermally responsive soft materials with stiffness‐shifting features, such as shape‐memory polymers (SMPs) and conductive elastomers (CEs), is their transition speed,^[^ ^12^, ^13^, ^14^ ^]^ as this feature is closely related to the thermal conductivity. Being poor thermal conductors, SMPs and CEs display low stiffness transition rates. In their solid state, LMPAs can serve as robust structural components in robotic systems due to their high stiffness in this phase (≈3 GPa).

In this paper, we capitalize the good processability at small scales and unique actuation features of LMPAs (i.e., high stiffness‐transition speed and high stiffness variation),^[^ ^15^, ^16^, ^17^, ^18^ ^]^ to develop a magnetically‐guided continuous VS (CVS) catheter. In contrast to conventional binary VS methods, the catheter continuously adapts its stiffness and provides a gradual change of stiffness.

**Figure 1:**
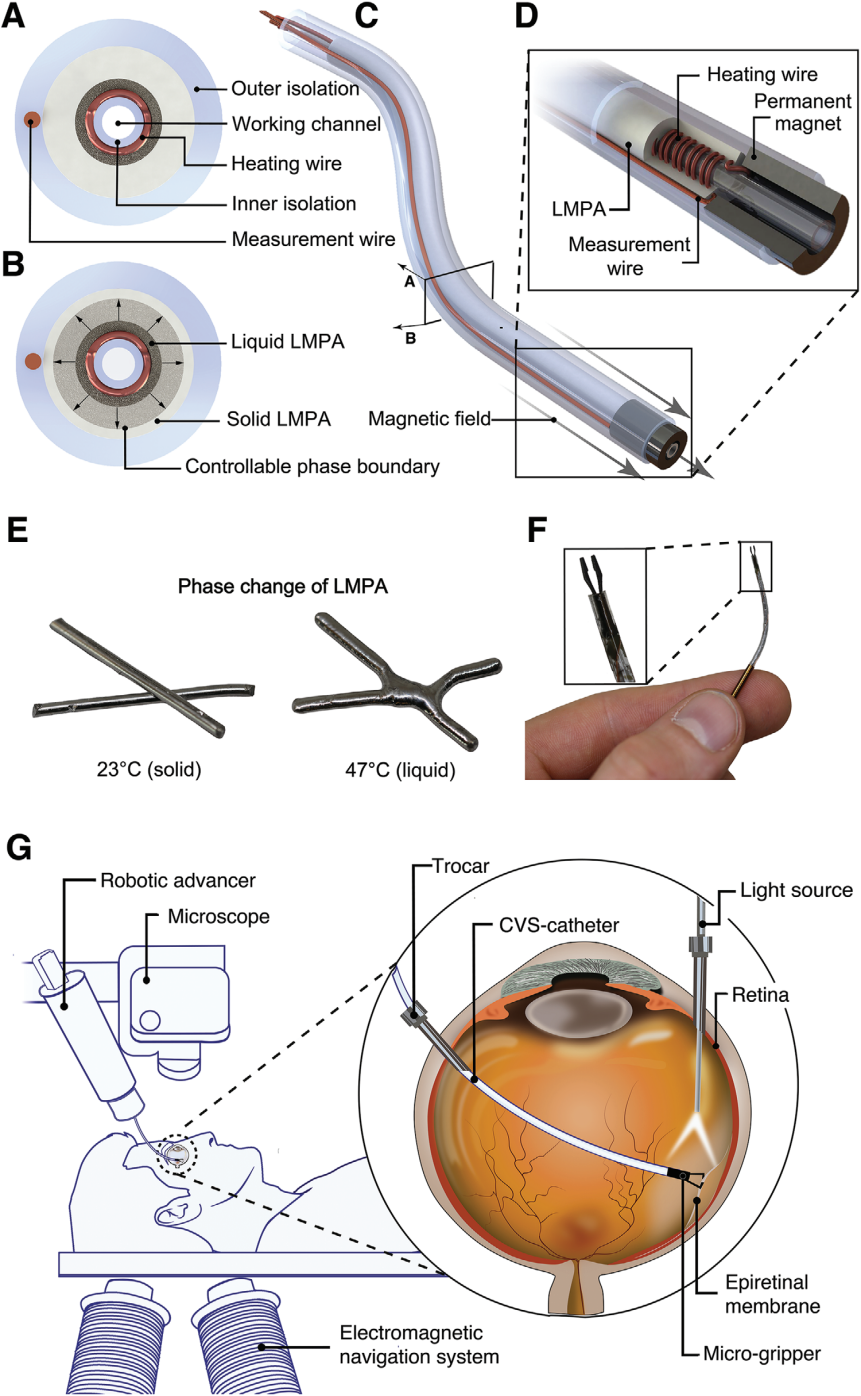
Schematic views of the continuous variable stiffness (CVS) catheter. A) Cross‐section of the catheter: the low melting point alloy (LMPA) core is encapsulated by an isolation layer and surrounds a working channel, and the heating and measurement wires are used for the control of the phase boundary within the LMPA. B) Control of the phase boundary: the radial heat expansion, due to the central heating and peripheral passive cooling from ambient air temperature causes the LMPA to melt from the inside out. Increased heat will shift the phase boundary radially outward. C) Overview of the proposed CVS catheter: the end‐effector tip has an embedded permanent magnet to steer the catheter with external magnetic fields. D) Close‐up view of the catheter: the heating wires are wrapped around the working channel, and the distal measurement wire is attached at the distal end of the LMPA and led back through the outer isolation. E) Non‐encapsulated LMPA cores before and after melting. F) The CVS catheter with a microgripper as an end‐effector. G) Clinical setup for a magnetically‐guided robotic membrane peel surgery: the magnetic navigation system is placed below the patient and allows the CVS catheter to be magnetically steered to the desired location. The compliance capabilities of the tool allow the surgeon to avoid tissue damage, as the catheter can be advanced with a robotic advancer that is placed above the patient. The procedure is monitored through a microscope, commonly used in most ophthalmic surgeries.

The catheter consists of a hollow working channel to deliver therapeutics or position instruments at the tip of the catheter. **Figure** **1** shows cross‐sectional views of the CVS catheter that reveal the design, components, and constituent materials. As can be seen in Figure 1A, the working channel is surrounded by a resistive coil that transmits heat to the surrounding LMPA (melting point of 47 °C), which is encapsulated in an insulating layer. We developed a scalable microfluidic approach to overcome the problems associated with conventional technologies to robustly fill extremely small sections of a catheter with a phase‐change alloy. This enabled us to manufacture solid, submillimeter diameter LMPA cores (Figure 1E). The phase transition temperature of the LMPA was carefully engineered. It must be above the working condition temperature (i.e., ≈37 °C, the temperature of the human body), but not too high to cause physiological damage. A radial temperature gradient in the device causes a phase boundary to be formed within the LMPA (Figure 1B). This phase boundary corresponds to a controlled radial temperature gradient that is achieved by placing the heat source in the center of the catheter. Numerical simulations were conducted to carefully design the CVS catheter accordingly (see Experimental Section for further details). For a low heat flux, the phase boundary is near the heat source. If the heat flux is increased, the phase boundary moves radially, and a higher proportion of the LMPA becomes liquid (Figure 1B). The volumetric fraction of solid‐to‐liquid LMPA is monitored with integrated wires that measure the electrical resistance of the material. Consequently, the current running through the heating coil can be adapted to achieve the desired overall stiffness. As shown in Figure 1C, the distal measurement wire is embedded in the outer isolation layer to minimize thermal effects that skew the electrical resistance. Additionally, the catheter can be guided by applying an externally generated magnetic torque to the permanent Neodymium Iron Boron (NdFeB) magnet, which is embedded at the tip (Figure 1D).

Magnetically guided tools have been explored for a variety of applications in cardiology,^[^ ^19^, ^20^, ^21^, ^22^, ^23^, ^24^ ^]^ interventional neuroradiology,^[^ ^25^, ^26^, ^27^ ^]^ ophthalmology,^[^ ^28^, ^29^, ^30^ ^]^ vascular surgery,^[^ ^31^, ^32^ ^]^ and gastroenterology.^[^ ^33^ ^]^ The coupling of VS tools with magnetic navigation has the potential to provide a new generation of advanced and smart surgical tools.^[^ ^11^ ^]^ None of these applications have included CVS technologies in their design. Our proposed approach, depicted in Figure 1F, is the first example of a continuum surgical device that elicits a controlled and adjustable stiffness and that can be steered via a hospital‐compliant electromagnetic navigation system (eMNS).

Our design can be used in MIS, where compliance control of interventional tools is critical to the outcome of the procedure. As an example, we use an eye phantom to perform robot‐assisted epiretinal membrane peeling with the CVS catheter (Figure 1G). The goal of epiretinal membrane peeling is the removal of a pathological cell layer that can form above the patient's retina and, if not removed, greatly impairs visual acuity. The retina is susceptible to physical damage that could be induced by the surgeon's hand tremor or unintended movements, as well as sudden patient movements.^[^ ^34^ ^]^ A functionalized catheter with adjustable compliance could therefore increase the safety and precision of the procedure.^[^ ^35^, ^36^ ^]^ To achieve this, the CVS tool, with an outer diameter of 1 mm, was equipped with an automated microgripper and tested on an eye phantom to perform robot‐assisted epiretinal membrane peeling. In its softest state, the purpose of the device is to navigate safely within the posterior eye to reach as many regions of the retina as possible while minimizing the risk of injuring tissues in case of contact. When we reach the desired area, the stiffness of the tool is increased such that we can apply forces to the tissue and close the gripper.

## 2 Results

### 2.1 Stiffness Control

**Figure 2:**
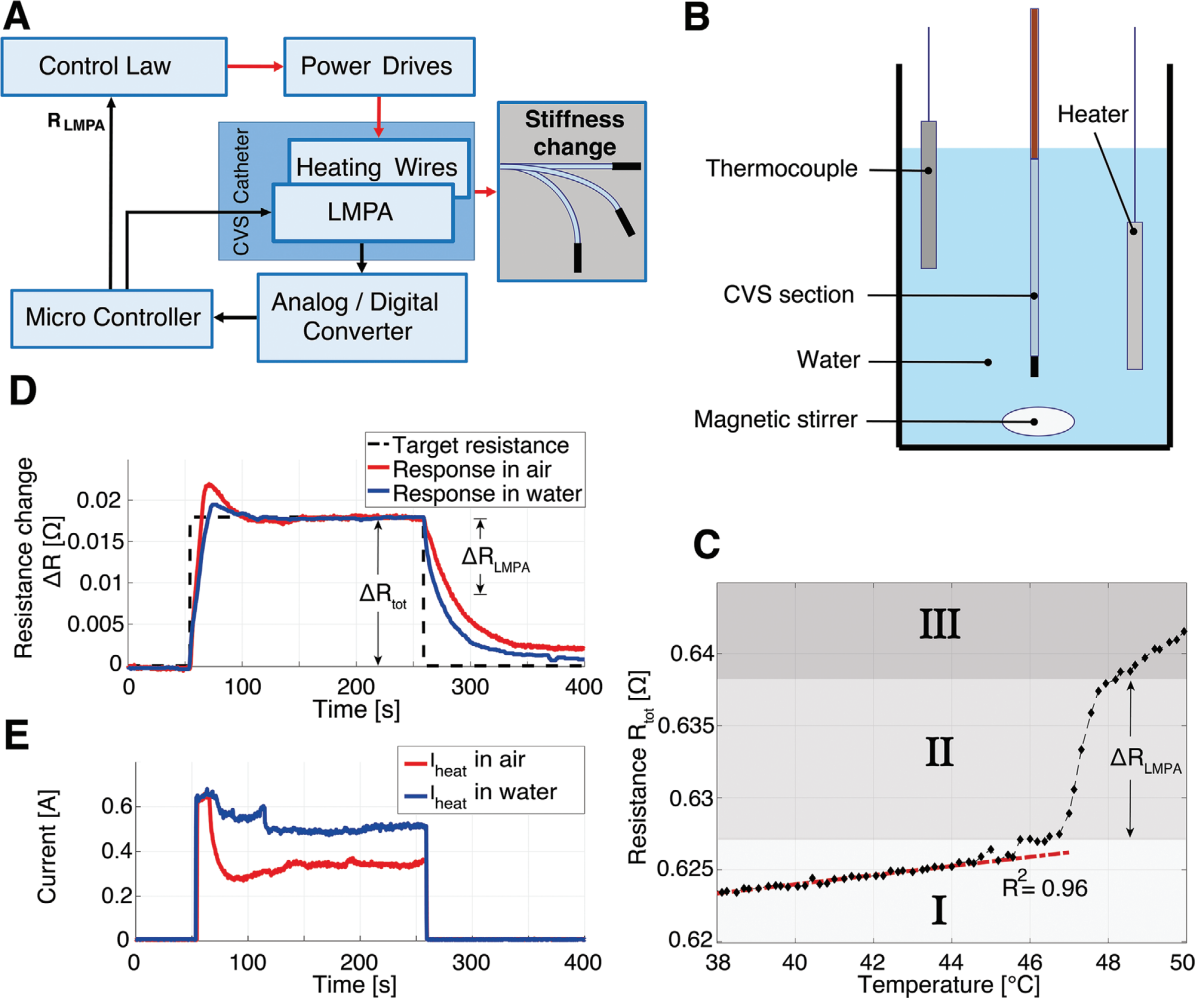
Control of the electrical resistance of the continuous variable stiffness (CVS) catheter. A) Signal flow to achieve stiffness changes in the catheter: the black arrows indicate the measurement loop, the red arrows the heating circuit. B) Schematic illustration of the setup for measurements for the temperature‐resistance relationship of CVS catheter. The catheter is immersed in a water tank with a controlled temperature; a magnetic stirrer guarantees a uniform temperature distribution. C) The resistance‐temperature relationship of the CVS catheter with the described measurement setup. Region I: Heating of measurement wires embedded in device; Region II: Phase change of LMPA; Region III: Heating of measurement wires and impurities D) CVS controller response to the desired resistance change from completely rigid to flexible in water (blue line) and air (red line). E) Applied electrical current by the controller to switch the CVS catheter from stiff to flexible in water and air, blue and red lines, respectively.

We designed a system to control the stiffness of the CVS catheter. **Figure** **2**A depicts the signal flow between relevant elements to achieve continuous stiffness control. The controller sends a signal to the power drives, which apply a voltage to the heating wires embedded in the CVS catheter. The heat produced by the resulting electric current melts the LMPA radially, as depicted in Figure 1B. In order to monitor the current state of the phase boundary, we capitalize on the fact that the LMPA has a higher specific electric resistance in the liquid than solid state.^[^ ^37^ ^]^ The variable resistance *R*_LMPA_ of the LMPA can be expressed as two resistors in parallel as follows:

(1) $$R_{\mathrm{LMPA}}=\frac{1}{\frac{1}{R_{L}}+\frac{1}{R_{S}}}\;$$

where *R*_L_ is the resistance of the liquid, and *R*_S_ is the resistance of the solid part of the LMPA. As the phase boundary expands outward and the solid material liquefies, the overall resistance (*R*_LMPA_) continuously increases. Thus, the phase boundary of the CVS catheter can be uniquely mapped to *R*_LMPA_.

To measure *R*_LMPA_, a small current was applied via the microcontroller to the measurement wires that are placed at the proximal and distal ends of the LMPA. Thus, we can measure *R*_LMPA_ indirectly through the total resistance (*R*_tot_) as

(2) $$R_{\mathrm{tot}}=R_{\mathrm{LMPA}}\;+R_{\mathrm{circuit}}$$

where *R*_circuit_ is the constant resistance of the rest of the measurement circuit. The total resistance *R*_tot_ can be obtained by monitoring the voltage and current between the wires. The signals were acquired using a voltage divider circuit with known reference resistances and an analog/digital converter (ADC) that can be read out by a microcontroller. The microcontroller calculates the overall resistance from the measured voltages and forwards the information to the controller. Proportional‐integral‐derivative (PID) control can be applied to achieve the users’ desired *R*_tot_ and the related stiffness change.

### 2.2 Temperature–Electrical Resistance Relationship

In order to investigate the relationship between the electrical resistance, the temperature, and the phase of the LMPA, the CVS catheter was placed in a controlled environment. As depicted in Figure 2B, a temperature‐controlled water tank was designed to equalize the temperature of the entire catheter so that no temperature gradients occurred within the LMPA. The overall resistance (*R*_tot_) was measured according to the measurement flow (black arrows) in Figure 2A. Figure 2C depicts the resistance, which increases linearly with the temperature until 47 °C (Region Ι), where the phase change of the LMPA occurs. The linear behavior at room temperature (coefficient of determination *R* ^2^ = 0.96) can be explained by the linear nature of the temperature‐electrical resistance relationship for electrical conductors. For the given catheter design, the complete transition from liquid to solid occurs in a band of approximately Δ*R*_LMPA_ = 0.01 Ω (Region ΙΙ). After the phase change, a linear increase in resistance with increased slope was measured (Region ΙΙΙ). Similar to Schubert et al.,^[^ ^12^ ^]^ the resistance continued to increase after the transition due to impurities in the LMPA material and heating of the measurement wires embedded in the CVS section. To achieve the maximum possible stiffness change in this device, we exploited the resistance change that occurred in Region ΙΙ due to the LMPA phase change as well as the slightly enhanced range of Δ*R*_tot_ = 0.018 Ω, which corresponded to a controlled temperature between ≈23–48 °C. Exploiting the thermal effects of both the LMPA and other components (including the polymer insulation and heating wires) allows for a larger range of stiffness control, while remaining within an acceptable temperature envelope (see Experimental Section—Requirements and Design).

### 2.3 Time Constants

Given the required resistance change (Δ*R*_tot_) to achieve a completely liquified CVS section from room temperature (23 °C), we evaluated the controller's capabilities, as depicted in Figure 2D. The figure depicts the actual resistance change (Δ*R*) over time to achieve Δ*R*_tot_, starting from the resistance at ambient temperature (23 °C) without any electrical heating. The catheter can be heated from a stiff to a flexible state in ≈10 s in air and 16 s in water. The settling time for this specific step response was observed to be around 1 min. Although the catheter took several minutes to cool down to its initial temperature, the resistance change responsible for the LMPA phase change (Δ*R*_LMPA_), and, consequently, the bulk of the stiffness change occurred after ≈17 and 30 s of cooling time for water and air, respectively. As depicted in Figure 2E, we applied just over 600 mA of current through the heating wires in the initial phase and required ≈450 and 350 mA of current to keep the catheter completely liquid in water and air, respectively. As the overall resistance of the heating wires is ≈1 Ω, the maximal power consumed amounts to less than 1 W.

### 2.4 Stiffness Characterization

**Figure 3:**
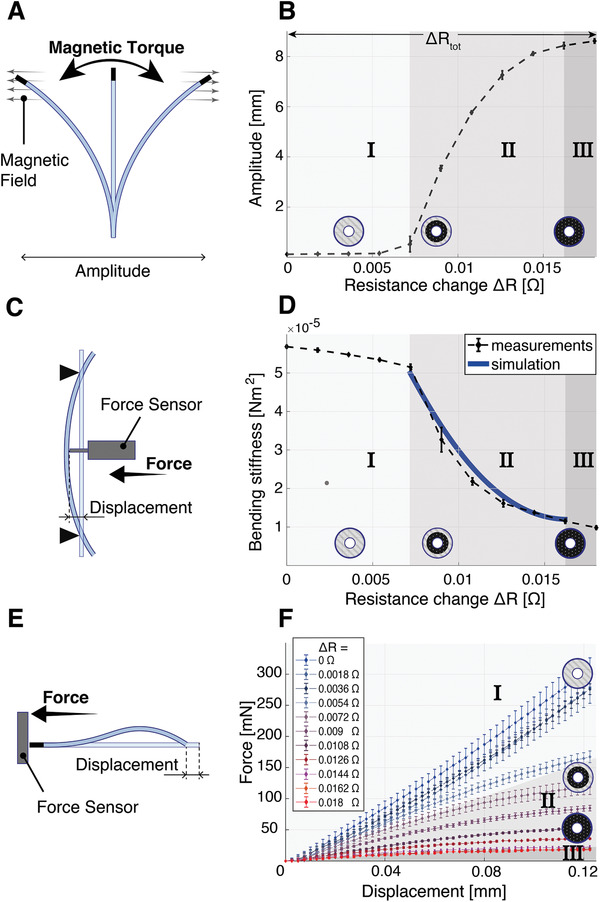
Characterization of the continuous variable stiffness (CVS) catheter. Dimensions: *L* = 30 mm, OD = 1 mm, ID = 180 µm. A) Amplitude measurements at different resistance states: a constant oscillating magnetic field of 40 mT was applied to the catheter with a magnetic manipulation system. The resulting oscillating magnetic torque deflects the catheter. Simultaneously, the resistance of the CVS catheter was controlled at discrete resistance levels and the obtained amplitude recorded. B) Oscillation Amplitude for a constant magnetic field and different controlled resistances (*n* = 10). The standard deviation at each measurement is depicted as an error bar. With the given magnetic field strength, the catheter does not oscillate if none of the low melting point alloy (LMPA) is liquid (Region Ι). As the LMPA liquefies, the amplitude increases nonlinearly (Region ΙΙ). When all of the LMPA has melted (Region ΙΙΙ) only a minor increase in oscillation amplitude was observed. C) Three‐point bending test setup. The catheter was fixed on two supports and a centered force deflected the catheter. From the resulting force–deflection curve, the bending stiffness could be measured for the same discrete controlled input resistances. D) Measured (*n* = 10) and simulated bending stiffness for different controlled resistances. E) Setup for compliance testing. The catheter was fixed on a linear stage and pushed toward a rigid force‐sensor. F) Force–displacement curves (*n* = 6) for the same set of discretely controlled input resistances. Due to bending of the catheter, the curves are concave, limiting the maximally applicable forces.

The controllability of the mechanical properties of the magnetic CVS catheter was characterized with three different setups. The length of the CVS section was 30 mm, the outer diameter of the catheter 1 mm, and the size of the working channel 180 µm. It was placed in an oscillating magnetic field (±90 ° offset from horizontal) with a magnitude of 40 mT and a frequency of 2 Hz, as seen in **Figure** **3**A. The catheter was fixed such that a 30 mm distal CVS segment was free to move. The externally applied magnetic field induced a torque on the magnet embedded in the tip. During the oscillation, the controller increased the stiffness in incremental steps by controlling the desired change in electrical resistance (Δ*R*) over the range of Δ*R*_tot_. The same set of discrete input resistances was used for all the experiments shown in Figure 3. The reference resistance for Δ*R* was chosen at ambient air temperature (23 °C) without any electrical heating. The qualitative variation in stiffness was detected by observing the change in the oscillation amplitude of the magnetic tip. The results presented in Figure 3B show that a stepwise increase in the desired electrical resistance results in a nonlinear increase in the oscillation amplitude. The regions Ι‐ΙΙΙ correspond to the respective regions in Figure 2C. These results clearly confirm that the stiffness change of the catheter can be physically determined and observed.

Additionally, the CVS catheter was subjected to a three‐point bending test to characterize the bending stiffness at the different controlled electrical resistances (see Figure 3C). The catheter showed a linear decrease in bending stiffness for lower resistances (Region Ι) due to the heating of the polymer components of the device (see Figure 3D). The subsequent nonlinear decrease in stiffness (Region ΙΙ) can be attributed to radial heat expansion and induced radial gradient in the LMPA. To model this stiffness variation from radial heat expansion, we can use Equation (2), which describes the total measured resistance *R*_tot_. Since the phase change occurs within 2 °C, we can omit the resistance change in the circuit due to temperature variations. Assuming a radially growing phase boundary (see Figure S1, Supporting Information), we can then express the phase boundary radius *r* as a function of the measured total resistance (*R*_tot_) as

(3) $$r\;=\sqrt{\frac{\frac{\rho_{S}\rho_{L}L}{\left(R_{\mathrm{tot}}-R_{\mathrm{circuit}}\right)\Pi }+{r_{i}}^{2}\rho_{S}-{r_{o}}^{2}\rho_{L}}{\rho_{S}-\;\rho_{L}}}\;$$

where *ρ*_*S*_ and *ρ*_*L*_ are the electric resistivity of solid and liquid LMPA, respectively, *r_i_* and *r_o_* are the inner and outer radius of LMPA, respectively, and *L* is the length of the VS segment. We assume that the materials in the catheter are bonded together, such that they are subject to an equal deformation. The contribution of the liquid LMPA to the overall stiffness of the device is negligible. The device's average Young's Modulus (*E*_avg_) can then be approximated by

(4) $$\begin{array}{ccc} E_{\mathrm{avg}}\left(r\right) & = & \frac{E_{\mathrm{LMPA}\;}A_{\mathrm{LMPA}}+\;E_{\mathrm{polymer}\;}A_{\mathrm{polymer}}}{A}\\ & = & \frac{E_{\mathrm{LMPA}\;}\left({r_{o}}^{2}-r^{2}\right)+\;E_{\mathrm{polymer}\;}\left({r_{p}}^{2}-{r_{o}}^{2}\right)}{\left({r_{p}}^{2}-r^{2}\right)}\; \end{array}$$

where *A*_LMPA_ is the area of the solid LMPA, *A*_polymer_ is the area of the isolation layer, *E*_LMPA_ and *E*_polymer_ are the Young's Modulus for the solid LMPA and the isolation layer, respectively, and *r_p_* is the outer radius of the isolation layer.

The moment of inertia of the LMPA (*I*_LMPA_(*r*)) is calculated by assuming an annulus with the phase boundary, *r* as the inner radius and *r_o_* as the outer radius

(5) $$I_{\mathrm{LMPA}}\left(r\right)=\frac{1}{4}\;\pi \left({r_{o}}^{4}-r^{4}\right).$$

To include the moment of inertia of the polymer (*I*_polymer_), we simplified the problem by converting the device to an equivalent system composed of a single material. We transformed the width of the isolation layer according to the modular ratio *n* = *E*_polymer_/*E*_LMPA_. The moment of inertia was then obtained as

(6) $$I_{\mathrm{polymer}}=\frac{1}{4}\pi \left({\left(r_{o}+\left(r_{p}-r_{o}\right)n\right)}^{4}-{r_{o}}^{4}\right).$$

The resulting bending stiffness change for a given electrical resistance can finally be calculated by

(7) $$K=E_{\mathrm{avg}}\left(r\right)\left(I_{\mathrm{LMPA}}\left(r\right)+I_{\mathrm{polymer}}\right):=\;F\left(R_{\mathrm{tot}}\right).$$

As both *E*_avg_ and *I*_LMPA_ depend on the current phase boundary radius (*r*) as defined in Equation (3), the estimated bending stiffness can ultimately be expressed as a function F of the total measured resistance. As depicted in Figure 3D, the simulation matches the measurements with a mean absolute error of less than 2%, allowing us to dimension CVS catheters that use LMPAs.

To demonstrate the catheter's capabilities for compliance, we placed it on a linear stage and pushed it toward a micro‐load cell (Figure 3E). The resulting force–displacement curves for a given controller setting for each set of experiments are displayed in Figure 3F. The results indicate that a single catheter is capable of displaying various compliance states (Movie S1, Supporting Information). The force–displacement curves exhibited a concave shape, allowing the catheter to limit its maximal force applied to a surface. The forces are limited due to the fact that the catheter bends and thus exhibits decreased axial stiffness. In the completely soft state (Region ΙΙΙ), the catheter can apply a maximum force of 20 mN. Figure S2, Supporting Information shows the relationship between large deformations and the corresponding compliance of the catheter. The force–displacement curves in Region Ι do not flatten out until they reach a peak of ≈8 N at a displacement of 1.1 mm of the catheter (Figure S2, Supporting Information).

### 2.5 Robotic Epiretinal Membrane Peeling

A prototype CVS catheter was specifically designed for ophthalmic surgeries, which are characterized by their minimally invasive nature and their need for compliance control. A tool that has a diameter of 1 mm that can also control and limit the forces that a surgeon can apply to the tissue could significantly improve current procedures.

Epiretinal membrane peeling is a delicate surgery that only highly skilled surgeons can perform, and was therefore selected as a procedure to validate our robotic tool. In this intervention, the surgeon manually removes a thin (≈61 µm) pathological layer that has formed on the retina.^[^ ^38^ ^]^ Before the tools are inserted, the eye vitreous is removed and replaced with a balanced salt solution (BSS) that has a similar viscosity to water. The eye vitreous is a gel‐like fluid that contains collagen fibers that can attach to the retina. Replacing the vitreous with BSS allows the surgeon to move the tools freely without creating sheer forces on the retina that could cause retinal tears or ruptures. Figure 1G shows the robotic procedure in a clinical setting. The standard setup of the ophthalmic surgeon is complemented by an eMNS that is placed below the patient to minimize obstruction. The eMNS can generate well‐defined magnetic fields at the position of the distal magnet to steer the catheter using current‐controlled electromagnets. To fully automate the procedure, a robotic catheter advancer (RCA) is mounted next to a standard surgical microscope. The surgeon can observe the procedure through the microscope and magnetically steer the CVS catheter to peel the epiretinal membrane with a handheld input‐device (not depicted, see Movie S2, Supporting Information). Thanks to its small diameter, the catheter can be inserted through a standard ophthalmic trocar (19 G). Surgeons typically use another trocar to introduce a light‐source for illumination. During the surgery, the catheter's compliance can be continuously adapted to avoid damage to the tissue, while ensuring sufficient contact forces between the gripper and membrane.

**Figure 4:**
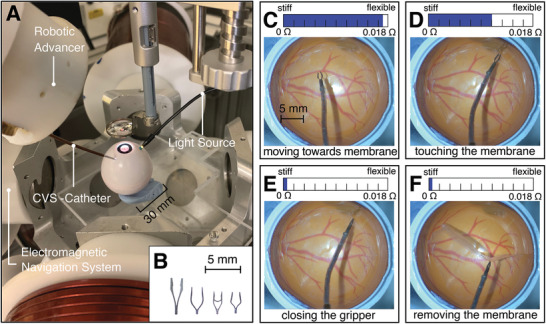
Robotic epiretinal membrane peeling surgery in an eye phantom A) Setup to validate a fully robotic epiretinal membrane peel intervention, consisting of the electromagnetic navigation system, the robotic colinear advancer, the continuous variable stiffness (CVS) catheter, a phantom eye, a light source, and a microscope. B) Custom microgrippers to perform epiretinal membrane peeling. C) The catheter can be moved efficiently in the flexible state to the location of the membrane. D) The intermediate stiffness level limits the forces that are applied to the tissue when making contact. E) To close the gripper, the stiffness is increased to minimize the position change due to the forces applied by the pull‐wire attached to the gripper. F) The membrane can be removed while the gripper remains closed.

This approach was evaluated using the OctoMag eMNS^[^ ^39^ ^]^ depicted in **Figure** **4**A. To perform the surgery, the CVS catheter was equipped with a custom microgripper fabricated by electrical discharge machining from a 300 µm thin feather steel plate (see Figure 4B). A 100 µm Nitinol wire was attached to the gripper using a laser‐welding process and inserted into the working channel of the catheter. When a pulling force on the wire was applied, the gripper closed as it was pulled into the distal ring magnet of the CVS catheter. As a result of the design and material, releasing the applied force automatically opened the gripper. The pull‐wire system was attached to the RCA, which is capable of moving the catheter and actuating the gripper simultaneously through its colinear design. A thin Parafilm layer was pressed onto the eye phantom, as this is acknowledged as an accurate model of epiretinal membranes.^[^ ^34^ ^]^

During the procedure, the CVS catheter was first completely flexible to facilitate maximal maneuverability of the tool (Figure 4C). As seen in Figure 4D, we approached the epiretinal membrane with the stiffness level set to an intermediate state (Δ*R* = 0.0108 Ω) to limit the maximum forces that can be applied below 100 mN (Figure 3F). This was estimated based on a study that measured the forces during epiretinal membrane peeling.^[^ ^36^ ^]^ As the catheter made contact with the surface, we moved along the given force–displacement curve with every step of the advancer. This allowed us to estimate the force currently applied to the membrane. For the given stiffness level, the catheter only bent marginally at the tip, facilitating a straight approach of the micro gripper to the surface. The stiffness should not be too low in order to reduce the bending of the tool, which could make gripping the membrane more difficult. Subsequently, the stiffness was increased further to close the gripper without incurring movement of the catheter (Figure 4E). No additional forces were added to the tissue from this stiffness increase. After grasping the membrane, it was successfully removed by pulling the stiff catheter back with the RCA (Figure 4F). The operation was controlled manually with a haptic input device that mapped the hand movements of the users to magnetic fields, and RCA commands that caused the tool to move to the desired location. The haptic input device was actively controlled to be weightless and to remove hand tremor. The precision was only limited by the resolution of the RCA (5 µm) and the manual input of the operator.

## 3 Discussion

Our magnetic CVS catheter changes its compliance to impose a relatively large range of forces during surgical interventions. This was achieved by inducing a radial temperature gradient in the phase change material embedded in the device. The same material was used to sense the state of the catheter, which allows accurate control of its stiffness and compliance. We showed that this concept is scalable down to millimeter‐sized tools, and is applicable to challenging surgical procedures such as in ophthalmology. We equipped the CVS catheter with a microgripper that can be actuated through the working channel. The catheter showed high dexterity in the flexible state and successfully resisted axial forces applied by the microgripper in the stiff state. During the operation, we were able to control the maximal forces that were applied to the tissue at all times. This allowed us to robotically peel a thin membrane from an eye phantom, simulating the removal of a pathological layer that can form on the retina.

The compliance depicted in Figure 3F can be tuned during the surgery according to the priorities of the operator. If there are concerns about unpredictable movements of the patient, the stiffness can be minimized. This leads to increased bending of the tool due to the contact forces. Conversely, the stiffness can also be increased if the contact with the membrane can be more reliably controlled. Assuming minor deformations of the tissue, the applied force can be estimated with the curves presented in Figure 3F. The chosen compliance depends on how these uncertainty factors are prioritized and should ultimately be the decision of the surgeon.

The phase change alloy used in this study has components that are not biocompatible, and it is crucial that the isolation layer remains intact under significant stress. Even though the catheter prototypes did not show any sign of rupture after many hours of testing in a lab setting, further efforts must be made to ensure the safety during an in vivo procedure. The risk could be mitigated by using different materials such as biocompatible polymers, where glass‐transition replaces the phase change of LMPAs.^[^ ^40^ ^]^

There is potential for CVS to further increase functionality and safety in catheters for various medical interventions. For example, navigating torturous pathways in the vascular system of the body is a challenging task. A CVS catheter with multiple sections would allow for a continuous change in stiffness along discrete sections of its body. A flexible distal section, which maximizes maneuverability at the tip, could be followed by a stiffer proximal section to minimize buckling.^[^ ^41^ ^]^ In contrast to current binary VS methods, the gradual change in stiffness of the CVS catheter can improve the insertion process and open pathways that could otherwise not be reached. Furthermore, CVS has great potential in applications that rely on a compliant interaction with the anatomy. Following the beating heart with a catheter tip in cardiac ablation procedures has been shown to be beneficial.^[^ ^42^ ^]^ With the proposed catheter, the stiffness could be set to optimally comply with the movement of the heart and apply adequate pressure.

This technology offers a solution to a fundamental challenge in medical robotics: surgical tools in the hands of robots must be dexterous, safe, and functional, however rigid tools are often limited to the latter. When combined with magnetic actuation, the CVS catheter overcomes these challenges, potentially improving the outcome of MIS and opening the field to novel interventions.

## 4 Experimental Section

### Magnetic Navigation

The externally‐applied magnetic fields were generated by the OctoMag eMNS, which uses eight electromagnets in a hemispheric arrangement to generate magnetic fields up to 40 mT in magnitude in any direction in space within a 10 cm‐side cube.^[^ ^43^ ^]^ The magnetic field strength and orientation were controlled by regulating the currents in each coil. A detailed description of the system design, mathematical description, and control can be found in.^[^ ^43^ ^]^ The CVS catheter was equipped with a permanent magnet at its distal tip such that magnetic torques modify the orientation of the tip. The torque (***t***) acting on the magnetic tip of the CVS catheter with magnetization (***m***) in [Am^2^] is given by ***t*** = ***m*** × ***b***, in [N m^−2^], where ***b***, in [T] is the applied magnetic field's flux density at the location of the magnetic tip. The magnetic tip tends to align in the direction of the applied magnetic field, which can be used to manipulate the catheter. The Phantom Omni (3D Systems, USA), a haptic device, was used to control the desired tool orientation. The encoder‐angles of the system were used to measure the pose of the hand‐held stylus, which could then be expressed in the frame of the magnetic navigation system (see Movie S2, Supporting Information).

### Requirements and Design

The CVS catheter was designed for eye surgery, as the size and temperature requirements were particularly restrictive in this organ, facilitating generalization to other applications.

**Figure 5:**
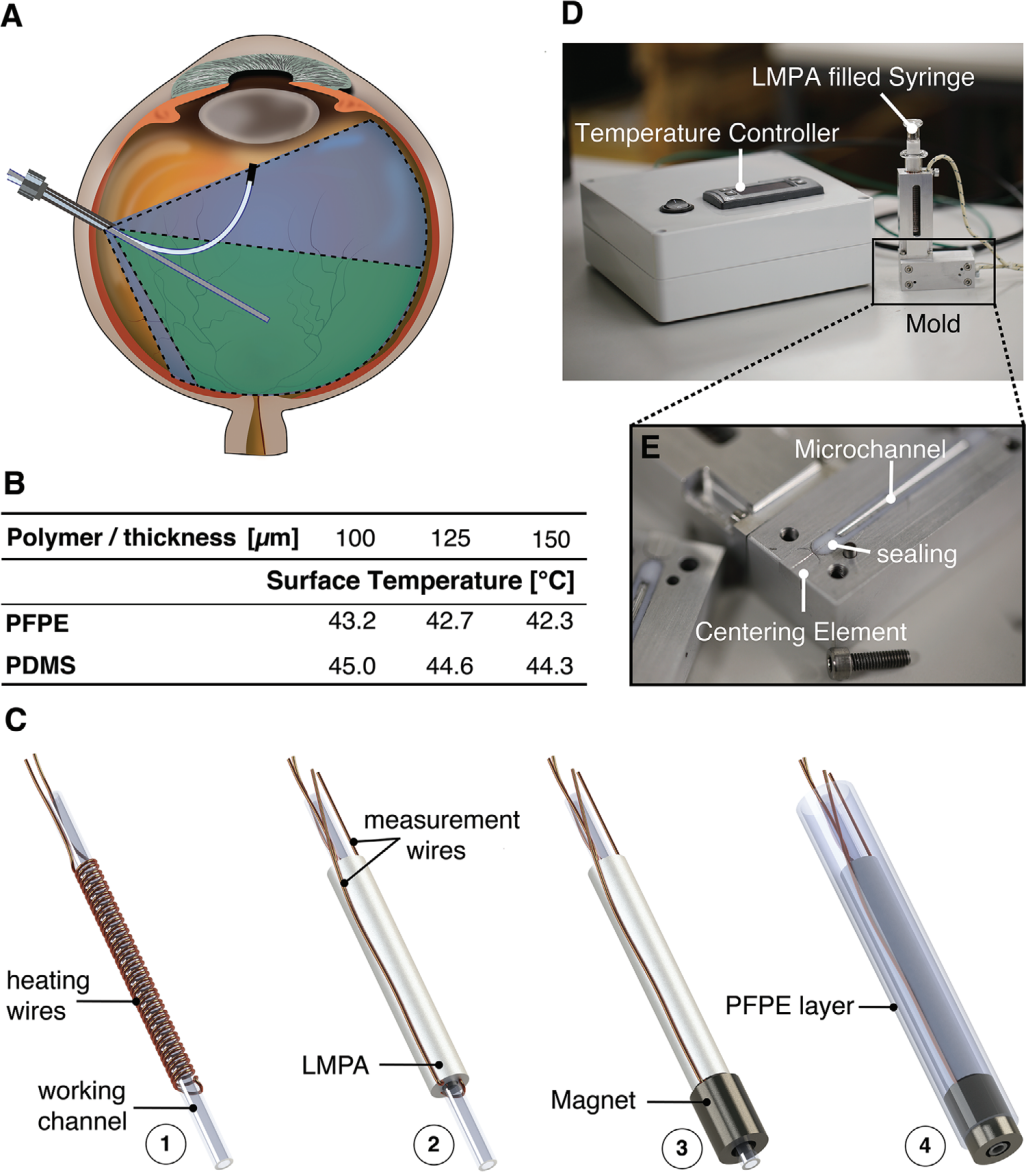
Design and Fabrication of the continuous variable stiffness (CVS) catheter. A) Workspace of a rigid and flexible tool: the workspace of a rigid tool is limited by the pivot point at the sclera (green area), a soft, magnetic tool has a potentially greater workspace and dexterity (blue area). B) Surface temperature for different polymer thicknesses. C) CVS catheter manufacturing steps: 1) after the heating wires are coiled around the working channel, 2) the Low Melting Point Alloy (LMPA) is uniformly coated around it. 3) The permanent magnet is then glued to the catheter. 4) Finally the device is coated with a 150 µm Perfluoropolyether (PFPE) layer. D) Mold and temperature controller for the fabrication of the CVS catheter. E) Close‐up of the microfluidic channels in the mold: the microfluidic channels are sealed with a teflon layer that is pressed together with screws.

In order to insert the CVS catheter into the eye and perform rigid manipulation thereafter, several design requirements had to be considered. First, the diameter of the tool should be as small as possible to allow for MIS. A tool that can be inserted in a standard 19 G (≈ø1 mm) trocar was designed. Second, in the soft state, the CVS catheter must be flexible enough to reach an acceptable workspace within the eye. Robotic systems that use traditional rigid tools for retinal surgery were able to reach a tool rotation range of up to 60° by pivoting the tool around the entry point at the sclera.^[^ ^44^ ^]^ In order to have a significant advantage compared to rigid tools, the minimal requirement for the tool rotation range was set to 90° for a 30 mm long CVS section. **Figure** **5**A illustrates the workspaces that can be reached with a traditional rigid tool (green area) and the flexible tool proposed in this work (blue area).

In the stiff state, the catheter must be able to support an axial force of 2 N required for gripping. This force was estimated using a setup where a wire pulls a customized spring steel gripper in a stiff 21 G needle in order to close it.

Furthermore, the catheter must satisfy the norms for temperature restrictions of foreign bodies inserted within the human eye.^[^ ^45^, ^46^ ^]^ If the temperature exceeded 41 °C, a risk assessment during a clinical study would be necessary. If the surface temperature reached 43 °C, procedures were also limited by time.

Finally, the LMPA (Cerrolow 117, RotoMetals USA; 44.7% Bismuth, 22.6% Lead, 19.1% Indium, 8.3% Tin, and 5.3% Cadmium) used in this study contains toxic elements such as Cadmium, and as such the catheter has to be encapsulated with a biocompatible material to avoid infection or allergic reactions.

### Material Selection

An LMPA of type Cerrolow 117 was selected as the material to be embedded in the catheter. Its mechanical characterization and use for VS applications using small soft structures has been previously published.^[^ ^11^, ^13^, ^37^ ^]^ In its solid state, it has a Young's modulus of ≈3 GPa, a tensile strength of 37 MPa, and an average strain at break of 3.2%. In the soft state, the LMPA exhibits a low stiffness and has characteristics of a liquid with low viscosity. Cerrolow 117 was chosen due to its optimal phase transition temperature (*T*_t_ = 47 °C). The phase transition temperature needs to be well above body temperature (37 °C) to enable rapid cooling for stiffness control. However, T_t_ has to be sufficiently low to minimize the thickness of the encapsulation layer required to reach the maximally allowed surface temperature of 43 °C.

The aforementioned requirements were carefully taken into account when designing the CVS catheter. The necessary isolation thickness to achieve a surface temperature of less than 43 °C was simulated in COMSOL Multiphysics. Perfluoropolyether (PFPE) was chosen as the outer isolation layer, as it has a significantly lower thermal conductivity (≈1/3) than the commonly used polymer for microfabrication. Polydimethylsiloxane (PDMS), while having a comparably low Young's Modulus.^[^ ^47^ ^]^ Figure 5B depicts the surface temperatures at different polymer thicknesses for PFPE and PDMS. The simulation assumed a 700 µm metallic rod in the center at 47 °C, surrounded by a variable polymer layer thickness that was subject to a surrounding volume of water at 37 °C. Standard heat transfer coefficients from the suppliers’ datasheets were considered. Due to the lower thermal conductivity of PFPE, the energy was better preserved within the LMPA and less heat had to be added, thus the surface temperature of the catheter was decreased. For the prototype, a 150 µm PFPE layer was chosen to limit the maximal surface temperature to well below the desired 43 °C.

### Fabrication

A novel and repeatable extrusion process was used to fabricate the CVS catheter, Figure 5C depicts the manufacturing process. In the first step (Figure 5C‐1), an electrically insulated copper wire (ø 50 µm) was coiled around a flexible polymer lumen (Tecoflex EG 65D B20, Microspec Corporation USA, OD = 300 µm, ID = 180 µm). The copper wire was used as the heat source required to control the stiffness of the LMPA. Based on the specific application, the polymer lumen can act as a tool channel for cable actuation, or for active cooling. The wire and the lumen were then coated with the LMPA (Figure 5C‐2) by placing it in a customized aluminum mold with a micro channel (Figures 5D and 5E). Centering elements were used to ensure a uniform thickness of the LMPA coating. The aluminum mold has inlet and outlet channels (ø 200 µm), which allow it to be flushed with liquid LMPA. The mold was sealed with a Teflon layer embedded around the micro channels. Heating elements and temperature sensors were incorporated within the mold. Due to the high thermal conductivity of aluminum, the temperature of the LMPA could be accurately controlled to optimize the filling of the micro channel. A set of insulated copper wires (ø 100 µm) were connected at the two ends of the LMPA. These wires were used to measure the overall LMPA resistance (*R*_LMPA_). A ring magnet (NdFeB N50H, X‐Magnets, China, OD = 900 µm, ID = 580 µm) was then placed at the distal end of the device and fixed in position with UV curable glue (Figure 5C‐3). Finally, the device was placed and centered in a glass tube (ø1 mm) and coated with a UV curable, biocompatible 4k‐PFPE layer (Figure 5C‐4).^[^ ^48^ ^]^ After the glass tube was removed, the proximal end of the device was covered with a protective tube to avoid damage to the measurement and heating wires.

### Controller Design

The controller consists of a custom printed circuit board (PCB) that connects a motor drive (drv8871, Adafruit, USA) and a 16‐bit ADC (ADS1115, Adafruit, USA) to a microcontroller. The measurement circuit was powered with 5 V and uses a reference resistance to determine the current *I*_LMPA_ flowing through the LMPA section. The measured voltage *V_LMPA_* in the CVS section can then be used to calculate $R_{\mathrm{LMPA}}=V_{\mathrm{LMPA}}/I_{\mathrm{LMPA}}$. The signals were passed through a second order analog low pass circuit before they were picked up by the ADC. The microcontroller communicates with a control computer through a USB connection using the robot operating system (ROS) at a frequency of 10 Hz. After receiving *R*_LMPA_, the computer calculates the necessary control input to the motor drives with PID control (*K*_P_ = 1, *K*_I_ = 0.4, and *K*_D_ = 0.4). The result was sent back to the microcontroller via the ROS message system and was transformed into an adequate pulse‐width‐modulated signal for the motor drives (more information in Figure S3, Supporting Information).

### Temperature–Resistance Characterization

Due to limitations in the experimental setup, parts of the heating wires that emerge from the proximal end of the CVS section were also exposed to the temperature‐controlled environment in Figure 2B. As a consequence, the measured resistance was artificially increased due to the effects of temperature on electrical resistance. In order to be consistent with the further characterization and calibration of CVS catheters, this effect was accounted for by applying a correction function Δ*R*_T_(*T*) on the measured resistance using

(8) $$\Delta R_{T}\left(T\right)=R_{23^{\circ }C}*\alpha *\left(T-23^{\circ }C\right)$$

where $R_{23^{\circ }C}$ is the resistance of the additional exposed copper wire section at room temperature, *α* is the temperature coefficient of copper, and *T* is the current measured temperature. The measurement curve *R*_tot_(*T*) displayed in Figure 2B was then obtained by applying the correction

(9) $$R_{\mathrm{tot}}\left(T\right)=R_{\mathrm{meas}}\left(T\right)-\Delta R_{T}\left(T\right)$$

where *R*_meas_ (*T*) is the raw measured resistance at each temperature step.

The water temperature was controlled at temperature intervals of 0.2 °C. A uniform temperature distribution in the catheter was guaranteed by introducing a measurement delay of 1 min. Each data point was averaged from 10 successive measurement samples acquired at 1000 Hz.

### Oscillation Amplitude Characterization

The oscillation amplitude was extracted by tracking the position of the tip on the video recording using blob detection.^[^ ^49^ ^]^ The raw data was filtered to account for the tracking noise. For each resistance level, five cycles were observed while increasing and decreasing resistance, resulting in 10 measurements per level.

### Bending Stiffness Characterization

To measure the bending stiffness, the catheter was fixed between two supports, allowing only linear motion, at a distance of 21 mm. A micro‐stage pushed a microforce sensing probe (FT‐S10000, FemtoTools GmbH, Switzerland) by *s* toward the catheter until a force *P* of 5 mN was reached. The derivative of the resulting force–deflection curve could then be used to calculate the bending stiffness *K* as

(10) $$K=EI=\frac{\partial P}{\partial s}\;\frac{L^{3}}{48}$$

where *L* is the length of the fixed catheter section. A line to the data was fitted to calculate the derivative. For each resistance level, three load deflection curves were measured while increasing and decreasing the resistance, resulting in 6 measurements per level.

### Compliance Characterization

To characterize the compliance, a linear stage (8MT167‐25BS, Standa, Lithuania) was pushed in 2.5 µm steps toward a micro‐load cell (3132_0, Phidgets Inc., Canada) until a 0.125 mm displacement was reached. This displacement limit was chosen to avoid plastic deformation of the CVS catheter in the stiff states, which could lead to a bias in successive measurements. The experiments were repeated ten times for each resistance level. The output of the micro‐load cell was measured with a 16‐bit ADC (ADS1115, Adafruit, USA) and synchronized with the corresponding microsteps through a microcontroller.

## Conflict of Interest

The authors declare no conflict of interest.

## Author Contributions

J.L. conceived the project, wrote the manuscript, and performed, together with M.M., the manufacturing, modeling, simulations, analysis, and experiments. S.S. designed the microfluidic mold, F.G. had an important role in programming the haptic device and setting up the experiments in the eye phantom. C.D.M. was responsible for the selection of the isolation layer, C.C., S.P., and J.P.L. directed the scientific process, Q.B. was essential in directing the research and designed, built, and programmed the temperature‐resistance setup. B.J.N. was the principal supervisor.

## Supporting information

Supporting Information

Supplemental Movie 1

Supplemental Movie 2

## Data Availability

The data that supports the findings of this study are available in the supplementary material of this article.
