# Supplementary material for: A Submillimeter Continuous Variable Stiffness Catheter for Compliance Control
Source: Adv Sci (Weinh). 2021 Jul 17;8(18):2101290. doi: 10.1002/advs.202101290 (PMC8456283; doi:10.1002/advs.202101290)
Supplement: Supplementary file 1 — Supporting Information [file ADVS-8-2101290-s001.pdf]

WILEY-VCH

© 2021 Wiley-VCH GmbH

ADVANCED SCIENCE
Open Access

Supporting Information

for *Adv. Sci.*, DOI: 10.1002/advs.202101290

A Submillimeter Continuous Variable Stiffness Catheter for Compliance Control

*Jonas Lussi, Michael Mattmann, Semih Sevim, Fabian Grigis, Carmela De Marco, Christophe Chautems, Salvador Pané, Josep Puigmartí-Luis, Quentin Boehler**, and *Bradley J. Nelson**

Supporting Information

A Submillimeter Continuous Variable Stiffness Catheter for Compliance Control

Jonas Lussi, Michael Mattmann, Semih Sevim, Fabian Grigis, Carmela De Marco, Christophe Chautems, Salvador Pané, Josep Puigmartí-Luis, Quentin Boehler, Bradley J. Nelson*

Supplementary Figures:

Figure S1. Scaled up model of radial phase boundary expansion

Figure S2. Impedance Characterization for large deflection of the Continuous Variable Stiffness (CVS) Catheter

Figure S3. Continuous Variable Stiffness (CVS) Controller Capabilities

Supplementary Movies:

Movie S1 (.mp4 format). Stiffness change demonstration

Movie S2 (.mp4 format). Demonstration of manual control, colinear advancer and epiretinal membrane peeling

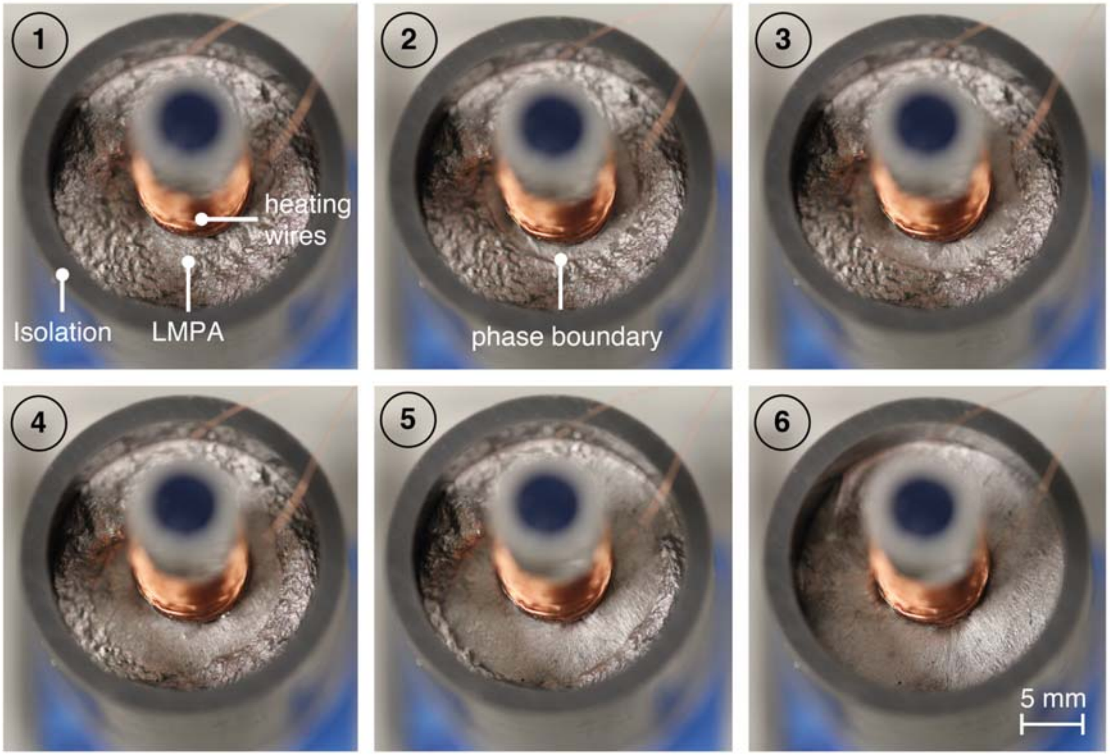

Figure S1. Scaled up model to illustrate radial phase boundary expansion. The scaled up model consists of heating wires that are located in the center of the device, Low Melting Point Alloy (LMPA) that is embedded around the wires and an outer isolation tube. Parts 1-6 show the qualitative expansion of the phase boundary for a strong, constant input current.

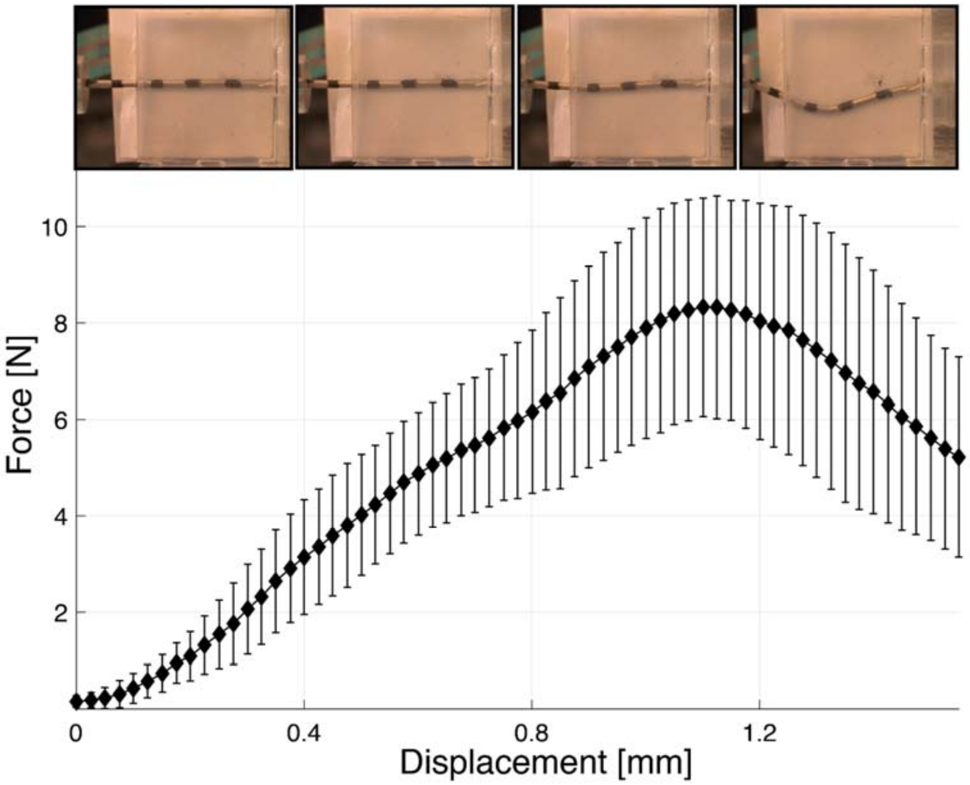

Figure S2. **Impedance Characterization for large deflection of the Continuous Variable Stiffness (CVS) Catheter.** The force-displacement curve is displayed for the completely rigid state of the CVS catheter. The experiment was done for 5 different prototypes. The average maximal sustainable axial force in the stiff state is 8.5N, with a minimum of 6N for the weakest catheter. Due to bending of the catheter and the associated decrease in axial stiffness, the force-displacement curve is concave.

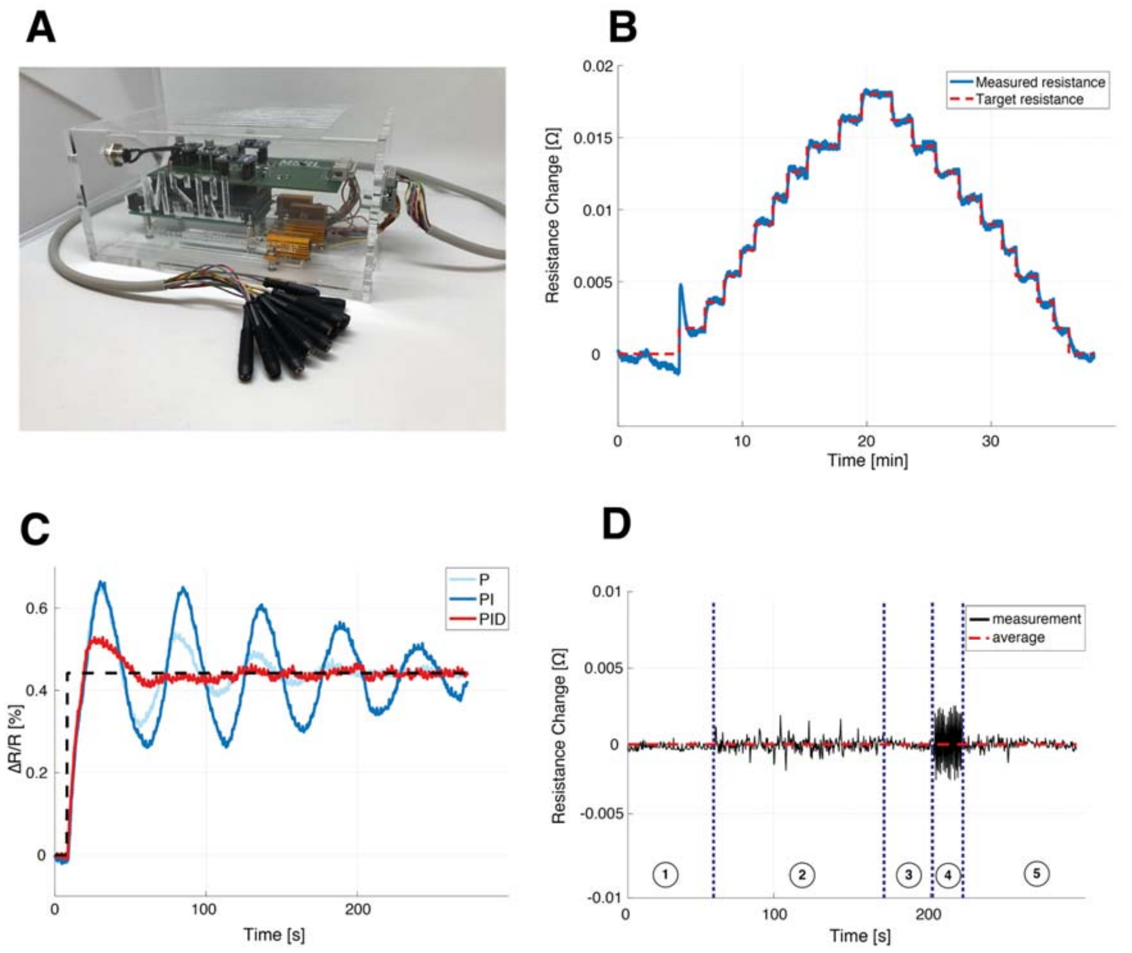

Figure S3. Continuous Variable Stiffness (CVS) Controller Capabilities. (**A**) Image of CVS controller box. The CVS controller is a compact box with PCB's, microcontrollers, analog-digital converters and motor drivers. It can control up to 4 sections simultaneously. (**B**) The 11 different controlled resistance levels that are also seen in the paper. The experiments were always done for both increasing and decreasing resistance. (**C**) The impact of adding different control terms (P – Proportional, I – Integral, D – Differential) to the controller. (**D**) Impact of disturbance on raw signal. (1) signal in standard conditions. (2) disturbance of randomly moving magnetic field. (3) forces are applied to CVS catheter by touching and moving it manually. (4) oscillating magnetic field is applied. (5) stationary magnetic field in arbitrary direction. Oscillating magnetic fields could reduce the resolution of possible stiffness levels.
